# Supplementary figures and images for: Adolescent hockey players’ predispositions to adopt sport and exercise behaviours: An ecological perspective
Source: PLoS One. 2020 Feb 14;15(2):e0228352. doi: 10.1371/journal.pone.0228352 (PMC7021282; doi:10.1371/journal.pone.0228352)

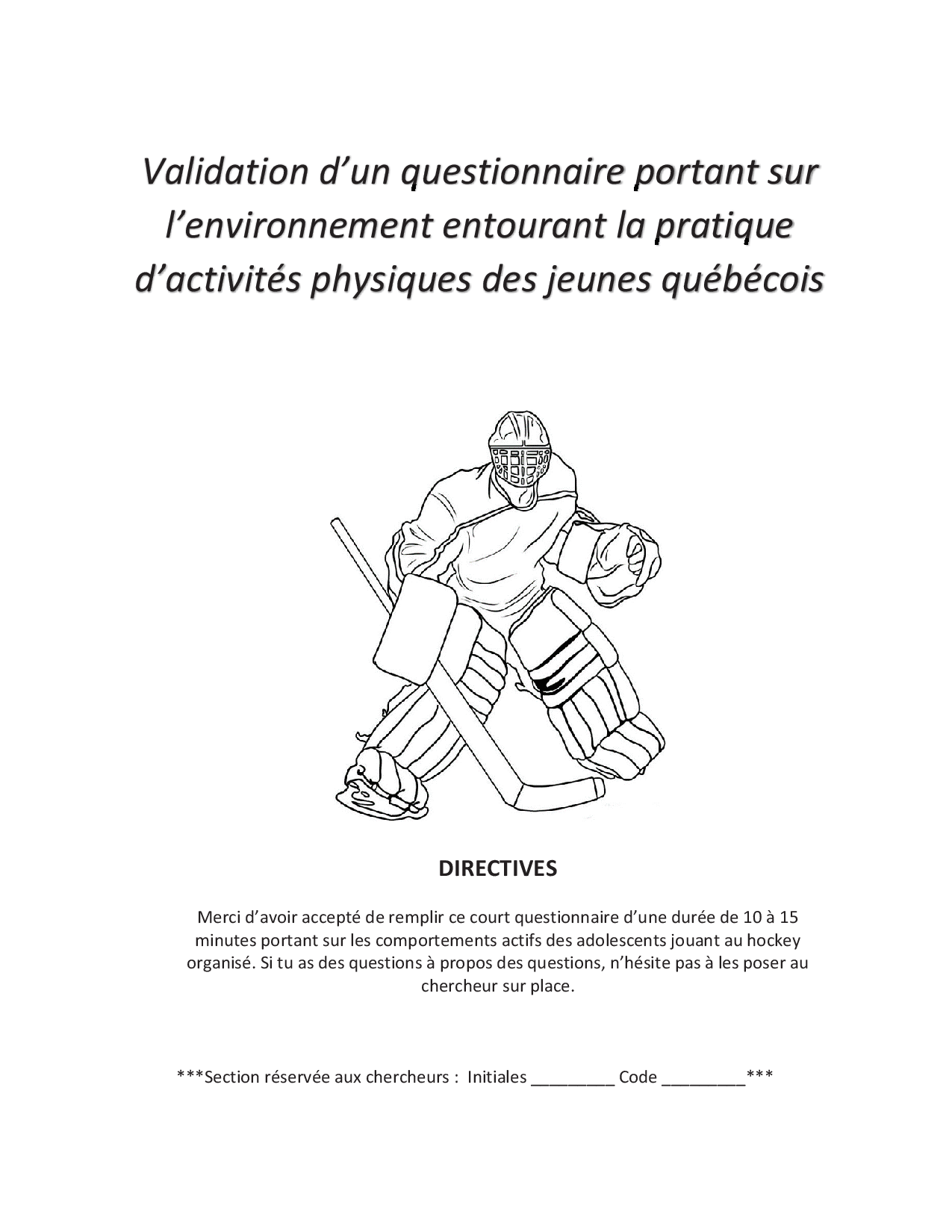

Supplement: S1 File — (TIF) [file pone.0228352.s001.tif]

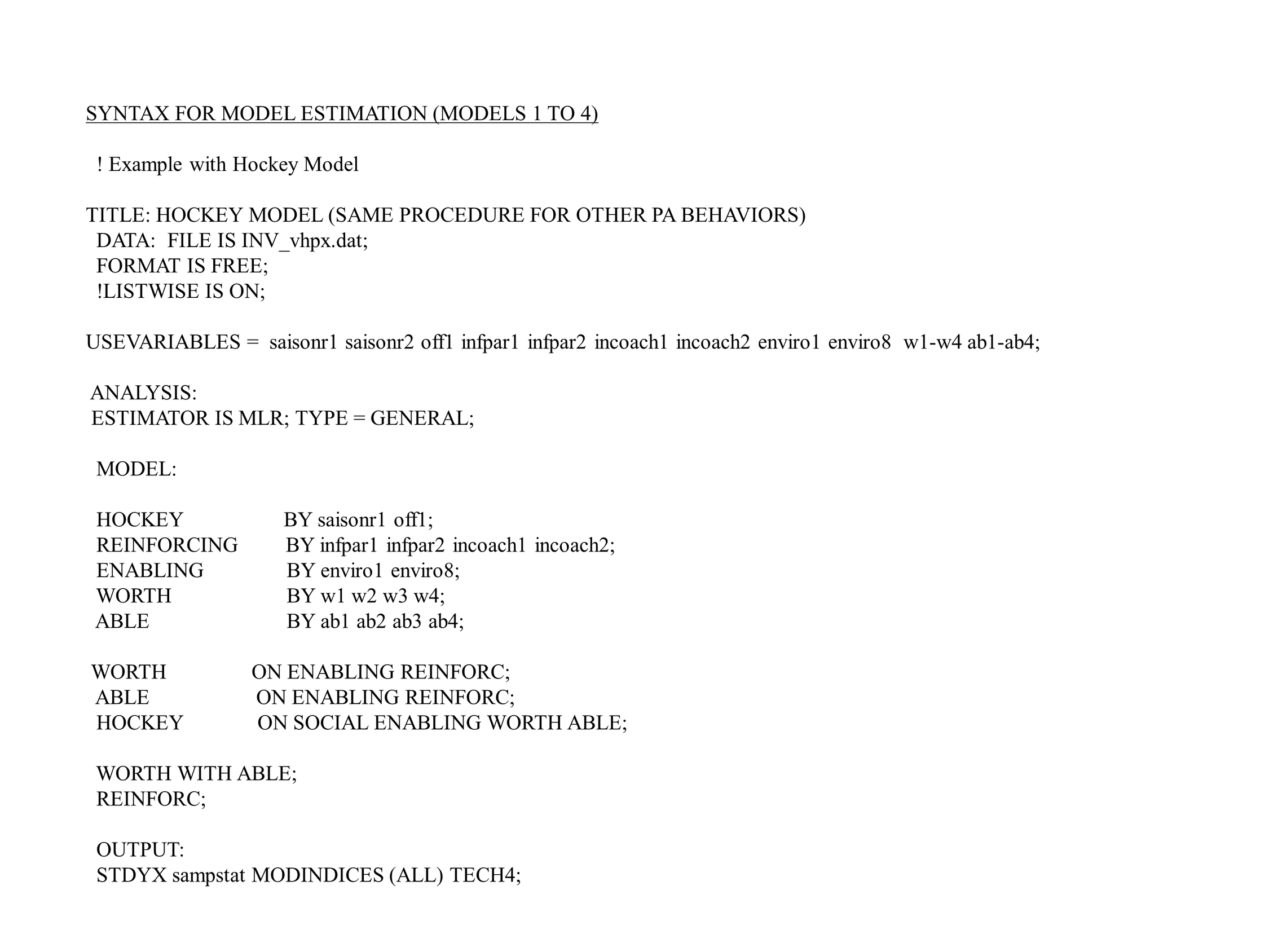

Supplement: S1 Fig — (TIF) [file pone.0228352.s002.tif]

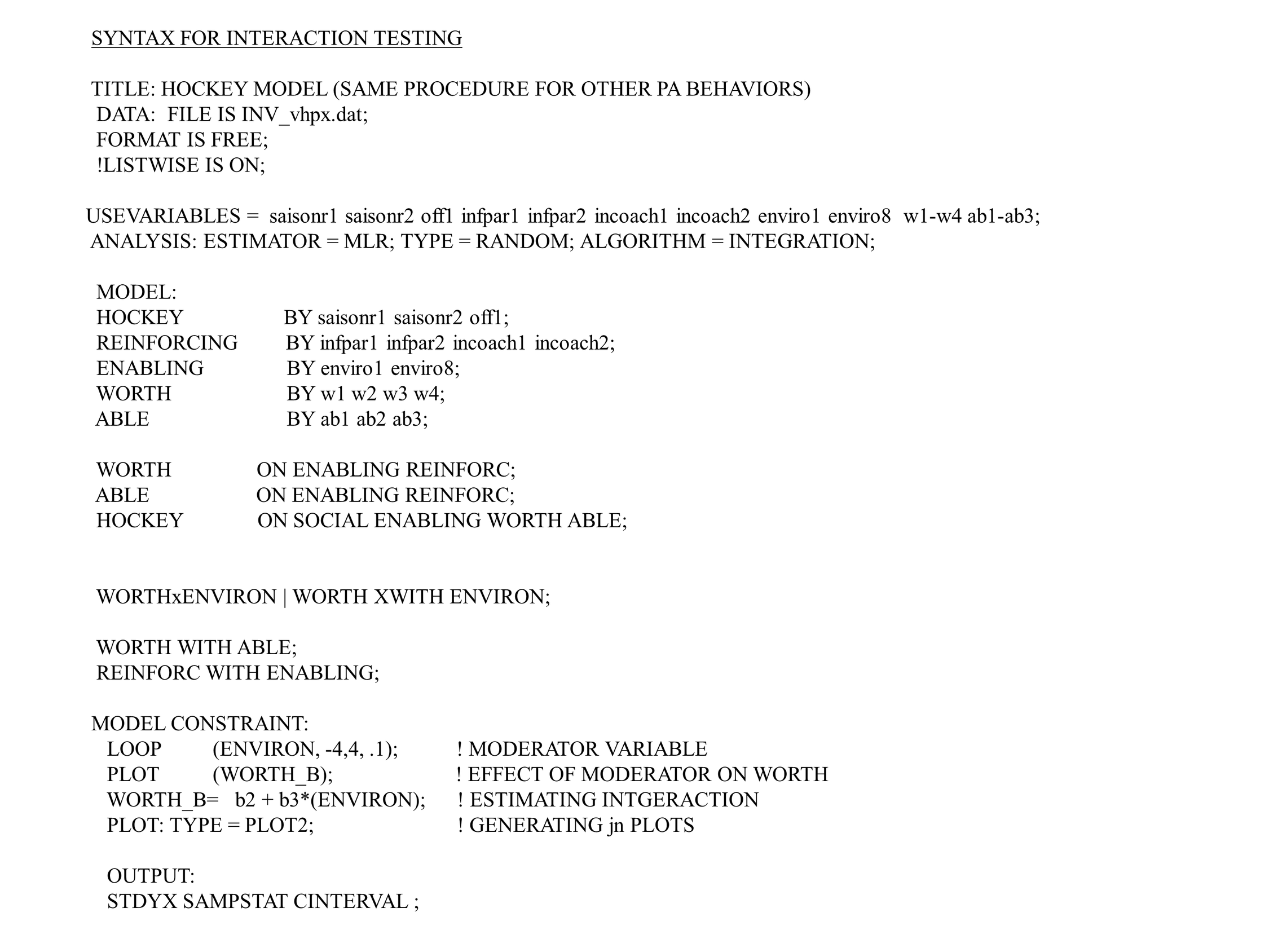

Supplement: S2 Fig — (TIF) [file pone.0228352.s003.tif]
