# Supplementary material for: Adolescent hockey players’ predispositions to adopt sport and exercise behaviours: An ecological perspective
Source: PLoS One. 2020 Feb 14;15(2):e0228352. doi: 10.1371/journal.pone.0228352 (PMC7021282; doi:10.1371/journal.pone.0228352)
Supplement: Attachment — Submitted filename: Authors response.docx [file pone.0228352.s004.docx]

| **Editor’s comments** | **Authors response** | **Modifications** |
| --- | --- | --- |
| Please ensure that your manuscript meets PLOS ONE's style requirements, including those for file naming. | Name files in numeric order with the format: Fig1.tif, Fig2.eps, and so on. |  |
| Method section: Please ensure you have provided sufficient details to replicate the analyses such as:  a) the recruitment date range (month and year),  b) a description of any inclusion/exclusion criteria that were applied to participant recruitment,  c) a table of relevant demographic details,  d) a statement as to whether your sample can be considered representative of a larger population,  e) a description of how participants were recruited, and  f) descriptions of where participants were recruited and where the research took place. | A and B) We added the recruitment dates (month/year), and a short sentence about our simple inclusion/exclusion criteria.  C) Demographic details are embedded in the text.  We added information about the a priori procedure to attain a sufficient level of statistical power to test our model.  D) No systematic sampling procedures were based, and we used a convenience sample for this study. However, efforts were made to try to get a representative sample on the proportions of participants in each age group and level of competition.  E) There was already some information in the text regarding the data collection, but we made sure to describe more precisely where the players filled the questionnaire. | P6: We used two strategies to collect data during the 2017-2018 hockey season  P6: All players between 12 and 17 years old, who were registered in the federation were eligible for the study  P22: The low proportion of female players was expected and can be explained. The 2% participation rate in our study is similar with data from the hockey federation regarding these age categories. ^4^  P6: A convenience sample was used for the study, but an effort was made to respect the proportions of participants at each level of play.  P6: The first questionnaires were collected by contacting members of elite hockey programs. Teams were identified via the website of Quebec’s ice hockey federation, and coaches were phoned or emailed to request permission to meet the players. The online version was filled in by the players at home while the paper … evolve before a practice.  P6: A second approach involved reaching out to hockey teams directly during tournaments. To … the teams at the registration desk. The directors gave the researchers permission to meet and inform the coaches, … or in their hotel if the research team was able to collect them back after the team’s final game. |
| Moreover, please include more details on how the questionnaire was pre-tested, and whether it was validated. | It was an omission to provide more details about the questionnaire elaboration phase. We added such information. | P7: “A questionnaire was constructed and pre-validated with a pilot sample consisting of 35 local young hockey players between 12 and 17 years of age in the months preceding the study’’. |
| If you developed a questionnaire as part of this study and it is not under a copyright more restrictive than CC-BY, please include a copy, in both the original language and English, as Supporting Information. | We only have a French version, that does not need CC-BY copyright. We attached a copy in the Supporting Information section. | See attached form in the Supplementary Files. |
| Figure 1. We require you to either (1) present written permission from the copyright holder to publish these figures specifically under the CC BY 4.0 license | Model’s author accepted to provide the signed copyright form. We are glad to see his interest towards the paper. | See attached form in the revised version. |
| Please amend the manuscript submission data (via Edit Submission) to include author MSc, Stephanie Girard | It was just an editing mistake Stephanie Girard is PhD. These corrections were made. | See Title page |
| Please include captions for your Supporting Information files at the end of your manuscript, and update any in-text citations to match accordingly. | We included a caption for the original version of our questionnaire. | P28: See at the end of manuscript. |

**Reviewer 1**

| **Reviewer comments** | **Authors response** | **Modifications** |
| --- | --- | --- |
| 1) Abstract: adjust numbers after comma. Insert the main results in the abstract. | We feel like the following sentences showed the main results of this study: ‘’Findings reveal that there are different behavioural patterns based on type of activity. …was associated with more recreational activities, whereas the support of parental and coaches determined involvement in ice hockey.’ |  |
| 2) Introduction: several phrases don’t have references. Please, add it. | We added some citations in the text while making sure we did not exceed too much the 40 references limit. | P3-P4: We added some citation that came from current citations. |
| 3) Methods:  Is the authors compared the responses of age rank, competitive level, and sexes?  Are the statistical analyses employed was enough to support the results? | Such interrogations are relevant, but not under the scope of the study. It is true that we mentioned competitive level and age groups (see P5), but the objective was to identify key predictors related with multiple active behaviors.  The *Competitive:Recreational* ratios was consistent with data obtained from our hockey federation. We decided to introduce some descriptive results regarding age and competitive level.  The analytic method suggested in this study is a solid option for path analysis. SEM approaches are ideal when the goal is to investigate relationships involving latent variables such as motivation, attitudes and other similar constructs. We added justification regarding the relevance of the method that we used. (see next page)  However, we did not analyze age and competition level as determinants of players’ engagement in active behaviors. Doing further analyses (path invariance according to age group or competitive level) would need larger samples, and would require other types of statistical analyses, such as invariance testing.  The analyses that were conducted (SEM with interactions testing) are certainly strong enough regarding the question research.  …  This is why we specified  (see P18-19) *Further analyses should be conducted to verify if the patterns displayed by more competitive players differ from those of recreational hockey players* | Some clarifications were added (see below)  …  P11-12: We verified for age and competitive level differences. Age group comparisons revealed that older players reported higher …, their perceived competence was not higher (t < 1.1; p > .10), excepted for perceived endurance: *t* = 2,12*; p* = .034.  P10: A structural equation modeling (SEM) path analysis approach was used to test relationships between YPAP model’s constructs. This approach has many advantages in the modelization of relationships involving latent variables^20^ |
| 4) Discussion: please, change in all information in the article the term “we”. Change to it was or other. | We have made the changes throughout the manuscript. | Minor corrections were made throughout the text. |
| 5) What the reproducibility of the fill these questionnaires? Are the authors measured it? | Du to time and feasibility issues, we decided not to conduct a repeatability test. The validation phase was conducted off-season, during a single occasion. A second measurement (for test-retest reliability) was not possible with this sample. | P7: A questionnaire was constructed and pre-validated with a pilot sample consisting of 35 local young hockey players between 12 and 17 years of age in the months preceding the study. Analyses were performed to assess each scale’s psychometric properties (Cronbach alpha and inter item correlations). Tests-retest reliability was not assessed due to the fact that it is difficult to gather a large number of participants during the off-season. |
| 6) The main point refers to aggregate all adolescents in a unique group. I believe that the groups should be split in competitive level and sex. | We agree with this comment, but the main focus here was to explore, for the first time, sources of influences towards active behaviors among adolescents, no matter the type of level in which they evolve. Investigating about age and the level of competition is certainly relevant, but would need important adjustments regarding sample size.  (See next page)  As mentioned in comment #3: *However, we did not analyze age and competition level as determinants of players’ engagement in active behaviors. Doing so would need larger samples, and would require other types of statistical analyses, such as invariance testing.* | No modifications.  P11: Except some descriptive statistics in the results section.  *We think that the justification in comment #3 is an appropriate answer to this comment.* |
| 7) Is the sample size for woman enough? Is represented the practitioners in Canada? | Obviously not.  An analysis of girls’ hockey would be a substantial addition to the research design because girls are playing in other leagues and tournaments at this age. We identify such as one of this study’s limitation. | P6: The lower proportion of female players was much lower due to many reasons. First, …girls categories play in female leagues-tournaments. …participation rate is similar with data from the hockey federation. |

**Reviewer 2**

| **Reviewer comments** | **Authors response** | **Modifications** |
| --- | --- | --- |
| This paper would benefit from some minor English language editing, as few grammatical errors were noted thought the manuscript. | We have done some language editing and it should be better now. | Some minor corrections were made throughout the text and are highlighted in yellow. |
| I ask the authors to consider a possible bias in the results based on the questionnaires collected in the second approach (during the tournaments). Having been the questionnaires applied at the end of the final game, could not have influenced some of the answers? Can this be considered a limitation of the study? | We forgot to specify that the questionnaires were filled during players free time (in hotel or in the arena) and they couldn’t fill them immediately before or after a match. We will add it to the text. It is also important to note that some questions, such as those related to physical activity and environment could hardly have been influenced by the outcome of the games. | P6-P7: ‘’The questionnaires had to be completed during the weekend in their free time, not immediately before or after a game. They were authorized to fill it in the arena or in their hotel as long as the research team was able to collect them back after the team’s final game.’’ |
| Results: Information is clearly provided on tables; however, the pictures appeared in the document with poor quality, hindering a more accurate visualization of the results. | Thank you for informing us. We will check with the editor to see if they are adequate or not. |  |
| Figures and tables: The three tables are clear and well designed. The three figures are with low definition impairing reading, especially figure 3. | Thank you for informing us. We will check with the editor to see if they are adequate or not. |  |
| References: There are 42, and all seem appropriate. | N/A | No changes |

**Reviewer 3**

| **Reviewer comments** | **Authors response** | **Modifications** |
| --- | --- | --- |
| It is suggested to rewrite the title, is big and not attractive. | We agree. | Modified title:  *Adolescent hockey players’ predispositions to adopt sport and exercise behaviors: an ecological perspective* |
| The abstract at some point does not make sense. First the authors expose that there is a substantial proportion of adolescents that don’t meet Canadian guidelines to have a good level of physical activity, and then the authors want to know more about the physical activity participation of adolescents already involved in an organized sport? | While the engagement in organized sports is related to an increased physical activity participation, it does not fully guarantee to fill the national physical activity recommendation. For example, a young hockey player who play 3 times a week is active for approximately 3-4 hours, which is not enough to be considered ‘’active’’. Moreover, in another article originating from this investigation, we found that only 40% of the players that were considered ‘’specializer’’ in ice hockey were fulfilling national recommendations during off-season. | Organized sport yields many cognitive, social and physical benefits and is one of the most popular types of physical activity for children and adolescents. Despite the benefits of sports participation, a substantial proportion of adolescents fail to meet Canadian guidelines regarding physical activity. In this regard, it is relevant to understand the mechanisms underlying the adoption of various active behaviours. |
| Why authors did not consider add girls to the study’s sample? | Due to practical and financial considerations, we chose not to include more female player in data gathering process. Less than 7% of the player in Quebec are female and there’s very few female tournaments so it would have been difficult to gather a large sample size at that time. | P21: The lower proportion of female players was much lower due to many reasons. First, we have to specify that every girls that was playing in the tournament was included in the assessment procedures. In fact, such rate of participation was expected, because girls categories play in female leagues-tournaments. In this regard, the 2% participation rate is similar with data from the hockey federation^4^ |
