# Supplementary material for: Adolescent hockey players’ predispositions to adopt sport and exercise behaviours: An ecological perspective
Source: PLoS One. 2020 Feb 14;15(2):e0228352. doi: 10.1371/journal.pone.0228352 (PMC7021282; doi:10.1371/journal.pone.0228352)
Supplement: Attachment — Submitted filename: Response to reviewers v2.docx [file pone.0228352.s005.docx]

**Response to Reviewer**

| **Section** | **Comment** | **Response** |
| --- | --- | --- |
| Table 1 & 2 | The authors should review the captions in tables 1 and 2, as they do not allow identifying some abbreviations. More specifically, in table 1, the abbreviations for “M” and “SD” are not defined in the caption. In table 2, are not identified in the caption, the abbreviations corresponding to the variables, which would facilitate the interpretation of the reader. | M= Mean, SD= Standard deviation. We added it to Table 1.  *p < .05 **p < .01 CFI= Comparative Fit Indice, TLI= Tucker Lewis Index, RMSEA= Root Mean Square of Approximation, R2= Coefficient of determination, χ²= Khi², df= Degree of freedom.  We added the same information to Table 2. |

**Response to Reviewer 3**

| **Section** | **Comment** | **Response** |
| --- | --- | --- |
| Introduction | In this section, it is considered that the pertinence of the study needs to be clearer. What is the real importance of the present study? | P5: The main objective of this study is to explore different mechanism that may influence adolescent already involved in organized sport to diversify their active behaviour. It’s a matter of importance because many of them will dropout of organized sports and thus may become sedentary in the future. Knowing which factor influence many types of PA can help the stakeholders that are promoting active lifestyle. We added a sentence saying essentially that just before stating the objectives of the study. |
| Methods | The authors should homogenize the terms that use all over the manuscript. In the introduction for the areas of physical activity used the following order and nomenclature: 1) organized ice hockey, 2) leisure-time sports, 3) weight training, and 4) cardiovascular training, however, in the variables and instruments, the authors change the order before and use different terms (e.g.: aerobic capacity). Please uniformize it. | We changed every ‘’cardiovascular exercise’’ to cardiovascular activities’’ through the text.  We also reorganized and respected the same order for the four types of active behaviours, especially in the *Methods* section. |
| Results | Table 1 need to be checked, there are wrong values (SD = 88 ?, or 1.700?), there also missing parenthesis. | **T1**: We corrected those values and added caption for Table 1 & 2. |
| Discussion | The authors mentioned in this section that previous studies mainly focused on younger participants (8-12 years old) but did not referenced. It is suggested to add those studies mentioned before. | **P16:** We added 2 references (Ahn & al., 2015; Seabra & al., 2013) but we can’t really add more du to the references limit set to 40 (we already have 47). |
| Perspective of future researches | The authors exposed that particular relationship has already been tested in some studies, but they only mentioned one study (Amorose et al., 2016). Please add more studies to justify the sentence. | **P21**: We added Mageau& Vallerand (2003) and Standage, Gillison, & Treasure (2007) to the references. |
